# Local Habitat Complexity and Its Effects on Herbivores and Predators in Urban Agroecosystems

**DOI:** 10.3390/insects15010041

**Published:** 2024-01-07

**Authors:** Azucena Lucatero, Shalene Jha, Stacy M. Philpott

**Affiliations:** 1 Environmental Studies Department, University of California, Santa Cruz, CA 95064, USA; sphilpot@ucsc.edu; 2 Integrative Biology Department, University of Texas at Austin, Austin, TX 78712, USA; sjha@austin.utexas.edu; 3 Lady Bird Johnson Wildflower Center, University of Texas at Austin, Austin, TX 78739, USA

**Keywords:** vegetation complexity, urban agriculture, predators, biological pest control

## Abstract

**Simple Summary:**

Garden plants provide habitat for pests as well as the predatory insects that eat them. In rural agriculture, structurally complex vegetation that includes diverse plant species can help attract predators and enhance pest control, while dense plantings of a single plant species are often associated with pest outbreaks. However, the vegetation composition that best supports predators and pest control in urban agriculture is not yet fully understood. In this study, we measured vegetation cover, diversity, and connectivity (distance between plants) in community garden plots to better understand how vegetation complexity influences the abundance and diversity of pest and predator species and pest control. Gardens with more vegetation cover had the most abundant and diverse predators, while gardens with more diverse vegetation had fewer predators. Further, gardens with abundant predators had higher pest control, whereas gardens with more predator species had lower pest control, possibly due to antagonistic interactions between competing predator species. Our results suggest that gardens with high vegetation cover can help support more predators, which can promote higher pest control. However, pest control benefits may be limited in gardens with diverse and antagonistic predator communities.

**Abstract:**

In urban community gardens, cultivated vegetation provides variable levels of habitat complexity, which can suppress pests by promoting predator diversity and improving pest control. In this study, we examine three components of the structural complexity of garden vegetation (cover, diversity, and connectivity) to investigate whether higher garden vegetation complexity leads to fewer herbivores, more predators, and higher predation. We worked in eight community gardens where we quantified vegetation complexity, sampled the arthropod community, and measured predation on corn earworm eggs. We found that plots with high vegetation cover supported higher species richness and greater abundance of predatory insects. High vegetation cover also supported a greater abundance and species richness of spiders. In contrast, high vegetation diversity was negatively associated with predator abundance. While high predator abundance was positively associated with egg predation, greater predator species richness had a negative impact on egg predation, suggesting that antagonism between predators may limit biological control. Community gardeners may thus manipulate vegetation cover and diversity to promote higher predator abundance and diversity in their plots. However, the species composition of predators and the prevalence of interspecific antagonism may ultimately determine subsequent impacts on biological pest control.

## 1. Introduction

Habitat complexity—the heterogeneity of biotic and abiotic components in an ecosystem—is an important determinant of the distribution and diversity of species [1]. The component measurements of habitat complexity can vary, but the structure and diversity of topography, substrates, and vegetation are frequently used as proxies [1,2]. Ecological observations have often linked high habitat complexity with greater species diversity and abundance in both aquatic and terrestrial systems [3,4,5,6,7,8]. This is because higher habitat complexity conferred by heterogenous vegetation and ground cover can provide a variety of microhabitats and microclimates, supporting a greater variety of species, functional groups, and foraging habits in arthropods [9,10]. The structural complexity of vegetation can also act as an environmental filter, shaping assemblages of arthropod species and their functional traits [11]. Specifically, structurally complex habitats can provide refuges and facilitate resource partitioning that supports the coexistence of predator and prey species as well as multiple competing predators in a system [12,13].

In agroecosystems, adding habitat complexity by diversifying crops and agricultural landscapes can promote the natural enemies of pests, leading to enhanced biological pest control [14,15,16]. Conventional agricultural practices typically produce simplified, monocultural habitats that often rely on pesticides to combat pest outbreaks [17,18]. In contrast, agroecological approaches to pest control emphasize biological diversification as a means of controlling pests [19,20,21]. Ecological theory backs several explanatory mechanisms for enhanced pest control in diversified systems. First, herbivores accumulate at high densities in monoculture plantings because of resource concentration of their preferred host plants, also known as the resource concentration hypothesis [22]. Crop diversification can therefore disrupt herbivorous pests by preventing them from finding host plants as well as reducing pest densities through bottom-up regulation [23]. Second, crop diversification can facilitate pest suppression through top-down regulation. According to the enemies hypothesis, complex habitats provide a variety of microhabitats, prey species, and alternative resources that support a greater diversity and abundance of natural enemy species [22]. In turn, a high diversity of natural enemies can result in greater pest suppression due to niche complementarity, whereby diverse natural enemy communities target different prey species or exploit different locations and thus consume more pests than assemblages with fewer natural enemies [24,25]. Empirical study of the relationship between habitat complexity and natural enemies generally supports the enemies hypothesis [26,27,28]. For instance, a meta-analysis examining 43 studies found that habitats with high vegetation and ground-cover complexity supported a higher abundance of natural enemies in seven out of nine natural enemy guilds [29]. More recently, a study of European vineyards found that increasing crop species diversity increased the abundance of natural enemies but had variable effects on predation rates [30]. Studies of the cascading impacts of habitat complexity on pest control are less common and deserve further investigation.

In particular, vegetation diversity is a key component of habitat complexity that likely has major consequences for biological pest control due to its diverse impacts on arthropods. The effects of plant species composition have received a great deal of attention in the natural enemies literature, with synthetic reviews of both rural and urban systems finding that plant diversity often promotes natural enemy diversity [23,31]. As addressed by the enemies hypothesis introduced above, plant species richness can benefit natural enemies through provision of diverse habitat and resources. Mechanistically, it is possible that a higher diversity of plants also results in a higher diversity of plant volatiles, the secondary metabolites emitted by plants in response to herbivory, which could recruit broad natural enemy groups via chemical signaling [32]. However, plant diversity effects on natural enemies can also be species-specific or depend on arthropod traits like size, diet breadth, and mobility [33,34]. In addition, vegetation diversity provides varied plant architectural structures, including variance in vertical height, branching structures, and size and shape of leaves and flowers [35]. Differences in plant architecture can affect arthropod mobility, search efficiency, foraging success, and mortality, with consequences for pest control [36,37,38]. For example, a study of four cruciferous plant architectures observing the foraging behaviors of predatory beetles found that variation in plant shape, texture, and surface area affected beetle foraging success by altering ease of movement, frequency of falling from the plant, and ability to reach aphids [39]. In some instances, architectural structures improved beetle grip on leaves as they foraged, while in others, architectural structures provided refuge for aphid herbivores from natural enemies. Plant architectural structures may thus advantage both herbivores and natural enemies, making it important to understand the exact conditions that favor natural enemy foraging success to inform agroecosystem management.

In addition to vegetation diversity, vegetation cover and connectivity are distinct components of habitat complexity with their own implications for biological pest control. First, vegetation cover, the percent cover of vegetation within a given quadrat or plot, can indicate the structural complexity of a habitat at small spatial scales [40]. Differences in vegetation cover produce different microclimates, such as zones with plants that are exposed to more sun in areas with low vegetation cover and zones with more shaded plants in areas with high vegetation cover. One study of urban greenspaces showed that light exposure decreased with vegetation structural complexity and that lower structural complexity decreased herbivore abundance, while light exposure increased abundance [41]. Considering the impacts of vegetation cover on natural enemies and biological pest control, studies have shown variable impacts. Vegetation cover and structural complexity were positively associated with predatory arthropod abundance in urban greenspaces [42,43,44], while studies of grasslands found that parasitism rates tend to be lower in habitats with higher vegetation cover, perhaps due to reduced host-finding ability under conditions of high structural complexity [36,40]. These variable results suggest that natural enemy foraging modes may determine the impacts of vegetation cover and structural complexity on pest control. Second, vegetation connectivity, or the spatial arrangement and overlap of plants relative to each other, can shape how arthropods navigate habitats [45]. A greenhouse study of ladybeetles on bean plants observed that ladybeetles traveled farther in treatments with high leaf overlap, suggesting that high connectivity between plants may increase predator foraging efficiency [46]. In the field, a study that artificially increased connectivity between coffee plants with string successfully increased ant activity and pest removal [47]. Examining the various facets of vegetation complexity can therefore reveal the mechanisms behind its impacts on biological pest control. 

Urban agroecosystems, such as urban farms and gardens, provide a unique opportunity to investigate the role of vegetation cover, diversity, and connectivity on pest control. Urban agroecosystems are dynamic greenspaces that provide diverse vegetative structures supporting urban biodiversity and ecosystem service provision [48]. Among these ecosystem service-providing species are the natural enemies of pests, which provision biological pest control through consumption of herbivorous pests. Biological pest control is a critical ecosystem service in urban agriculture [49,50] where the vast majority of urban growers report facing significant challenges in managing crop pests and often lack the technical knowledge to do so [51]. Urban community gardens are particularly well suited to investigating the components of vegetation complexity at the local scale. Community gardens are managed by multiple gardeners in individual allotment plots which vary in vegetation complexity depending on gardeners’ choice of cultivated plants and management practices like frequency of weeding and pruning [52]. At a larger, garden-site spatial scale, previous work in urban agroecosystems documented impacts of garden size, woody vegetation, floral abundance, and ground cover on herbivore abundance, natural enemy composition, and pest control [53,54,55]. The present study builds on this work by focusing on fine-scale vegetation complexity, specifically vegetation cover, diversity, and connectivity, and their impacts on arthropods within gardener plots. This is the true scale at which gardeners make changes to vegetation management and is thus a highly relevant spatial scale to investigate in these agroecosystems.

In this study, we quantify three key components of vegetation complexity (cover, diversity, and connectivity) in community garden plots and their subsequent impacts on the arthropod community and predation. We investigate two main questions: (1) Do these components of vegetation complexity differentially influence the abundance and richness of herbivores and predators at the garden-plot level? (2) How do these components of vegetation complexity affect predation levels provided by predators? Drawing from the resource concentration and enemies hypotheses, we predict that gardens with higher vegetation cover (higher percent cover of all plant species present in plots) will have more herbivores and lower predation levels, while gardens with higher vegetation diversity and connectivity will support more predators and enhance predation.

## 2. Materials and Methods

### 2.1. Study System

This study took place at eight community garden sites in the California central coast (Santa Cruz and Monterey counties) during the summer growing season in 2019 (1–6 August). We were limited to these eight sites (out of 30 gardens we previously studied in the region) because of constraints in recruiting gardeners willing to have their plots intensively sampled for the duration of this study. The garden sites ranged in size from 444 m^2^ to 6070 m^2^, were separated from each other by at least 2 km, and varied in both garden management and urban landscape context. Six of the eight garden sites were managed in allotments, where land is divided into parcels and assigned to individuals. The remaining two sites were managed collectively by student groups and school personnel. All gardens were organically managed and had been cultivated between 4 and 37 years at the time of this study. Gardeners typically grow a range of vegetables, fruits, herbs, and ornamental plants in their plots. Each site is located in the Monterey Bay Plains and Terraces ecoregion, which has a cool climate influenced by its proximity to Monterey Bay [56].

### 2.2. Vegetation and Ground Cover Surveys

At each garden, we collected data from four gardener plots (*n* = 32 plots), which ranged from 0.98 m^2^ to 70.3 m^2^. We first asked gardener permission to survey their plots for this study. From those plots offered at each site, we selected two plots that broadly represented low vegetation complexity (e.g., few plants) and two that represented high vegetation complexity (e.g., many plants) based on visual estimation. Low vegetation complexity plots contained 45% plant cover and 6 plant species per plot on average, and high vegetation complexity plots had 78% plant cover and 10 plant species on average. We then quantified the vegetation and ground cover in a 1.5 × 1.5 m area within each gardener plot to standardize the sampled area. Within each 1.5 × 1.5 m plot, we identified all plant species, estimated the percent plant cover, and measured the longest distance between two plants in the plot. An experienced member of our field team used PVC tube quadrats and tape measures to visually estimate the percent area of the plot covered by either vegetation or various ground cover types (e.g., bare soil, rocks, leaf litter, mulch, straw) (as per [57]).

### 2.3. Arthropod Community Surveys

We sampled the arthropods at each of the 32 sample plots twice within three days (on 1 August and 3 August). To sample arthropods on foliage, we haphazardly placed two 0.25 m × 0.25 m quadrats in each plot. We then visually surveyed the plants in each quadrat for up to five minutes by carefully inspecting all plant leaves and structures and recording the abundance and identity of all herbivore and predator species we encountered (similar to [57]). We counted but did not collect arthropods that were easily identifiable in the field and occurred at high densities (e.g., aphids, ants). Risk of double-counting arthropods over the two sampling periods was minimal due to haphazard placement of quadrats as well as human disturbance of gardener plots for research and garden caretaking. We collected all other arthropods and preserved them in 70% ethanol. To sample ground-dwelling arthropods, we placed one pitfall trap in the center of each gardener plot. Pitfall traps consisted of 12 oz. clear plastic containers filled halfway full with saline solution and a drop of detergent (as per [58]). We buried pitfall traps level with the surface of the soil and left traps in each plot for 24 h. After collecting the pitfall traps, we rinsed arthropod samples in water and preserved them in 70% ethanol. We identified all arthropods to morphospecies using dichotomous keys and online resources [59,60,61]. 

### 2.4. Sentinel Pest Removal Experiment

We conducted two rounds of sentinel pest removal experiments (on 2 August and 5 August, concurrent with the two rounds of arthropod sampling) to measure the predation services provided by predators occurring in each of the 32 study plots. We used potted fava bean (*Vicia faba*) plants grown under greenhouse conditions and inoculated plants with corn earworm eggs (*Helicoverpa zea*). We purchased eggs from Frontier Agricultural Sciences (Newark, DE, USA) under USDA-Aphis Permit P526P-14-02660. We chose corn earworm eggs because of their commercial availability and similarity to common prey items in our study sites (e.g., lepidopteran eggs of *Pieris rapae*, *Trichoplusia ni*, *Plutella xylostella*, and *Spodoptera exigua*, all of which are major garden pests as larvae). While corn earworm eggs are not herbivores per se, they are a convenient proxy that many arthropod predators prey on. Recent observations from our system confirmed that a variety of arthropod predators, including ants and predatory hemipterans, actively and swiftly (within a few hours of sentinel plant placement) remove egg prey [62]. To prepare egg prey, we cut the cloth sheets onto which eggs were laid into 1 cm x 1 cm squares (~600 eggs per square on average) and stored eggs in a freezer prior to field experiments (as per [54]). We randomly assigned egg squares to a site and field treatment, and we photographed all squares with a microscope camera before and after field experiments so that we could compare photos to determine the number of eggs removed during the experiment. In the field, we haphazardly placed one fava plant on the interior perimeter of each sample plot (within ~30 cm of the edge). We selected fava plant locations that were unobstructive to prevent damage to research plants from passersby. We then pinned egg squares onto two leaves per fava plant, bagging one leaf with a mesh paint strainer secured by a hair tie (predator exclusion treatment) and leaving the second leaf open (predator access treatment). After 24 h, we retrieved plants to collect egg squares for recounting. Previous work showed that 24 h is sufficient for significant predation to occur [54], and this short time window minimizes the risk that sentinel plants will be damaged or removed by passersby. Because eggs were frozen prior to the experiment, we assume any missing eggs did not eclose and were removed by predators (or lost during transport and setup, which we accounted for with the control, unbagged treatment). In total, we deployed 64 sentinel plants across all plots and both sampling rounds.

### 2.5. Data Analysis

For our predictor variables, we calculated three different metrics of vegetation complexity: cover (percent plant cover), diversity (number of plant species), and connectivity (1/the longest distance between two plants in a plot). In examining Pearson correlation coefficients between our vegetation metrics, we found relatively weak correlations (between 0.1 and 0.55; Table S1), indicating that these metrics captured distinct effects of vegetation complexity. Gardener plot size was also not significantly correlated with any vegetation complexity metric (Table S1).

For our response arthropod data, we pooled abundance, richness, and predation data over the two sampling rounds we conducted. We did not pool arthropod data from visual surveys and pitfall traps because these sampling methods occurred over different time spans (five minutes for visual surveys and 24 h for pitfall traps) and largely captured distinct foliage-level and ground-level foragers (80% of all morphospecies occurred only in visual surveys or pitfall traps). For response predation data, we used the log response ratio (LRR) as our effect size for moth egg predation, calculated as LN(proportion eggs removed in open treatments)—LN(proportion eggs removed in bagged treatments). We conducted all analyses in R version 4.2.1 [63].

We constructed four different groups of generalized linear models (GLMs) testing vegetation cover, diversity, and connectivity as predictors of the abundance and morphospecies richness of (1) herbivores, (2) predators, (3) the two most abundant predator taxa (ants and spiders), and (4) egg predation. We used the ‘Dharma’ package in R to visually assess standard residual and QQ plots and determine the best error distribution for each response variable [64]. We assumed a Poisson error distribution for herbivore richness, foliage-level predator richness, ant richness, and spider richness models. We fit models of herbivore abundance, predator abundance, ground-level predator richness, ant abundance, and spider abundance with a negative binomial error distribution to account for overdispersion. For the effect size of egg predation (noninteger values), we used a Gaussian error distribution.

For all model sets, we included vegetation cover, diversity, and connectivity as predictor variables. For models of egg predation, we included predator abundance and species richness as additional predictors. We used the variable inflation factor (VIF) to check for collinearity among all predictor variables using the ‘car’ package [65], and all VIF scores were under 2.4. We did not include site as a random effect because doing so resulted in singular models (indicating overfitting) [66]. Models with and without site as a random effect produced the same qualitative result (see Table S3 for generalized linear mixed model results), and both versions of models produced similar AICc values (within five points). We tested all combinations of predictor variables and selected the top model based on AICc values. When the top model was within two AICc points of the next model, we averaged models using the ‘MuMIn’ package [67].

## 3. Results

In total, we found 502 herbivores representing 11 families and 17 different morphospecies and 716 predators from 23 families and 35 morphospecies (Table S2). We recorded between 125 and 430 arthropods at each site. The most common herbivore families were Aphididae (*n* = 323), Aleyrodidae (*n* = 113), and Cicadellidae (*n* = 38). Formicidae (*n* = 532) was the most abundant family of predators, while the most diverse group of predators was the order Araneae (*n* = 13). Approximately 80% of morphospecies were sampled only through visual surveys or pitfall traps, indicating distinct arthropod foraging groups occurring on foliage or ground, respectively.

**Figure 1:**
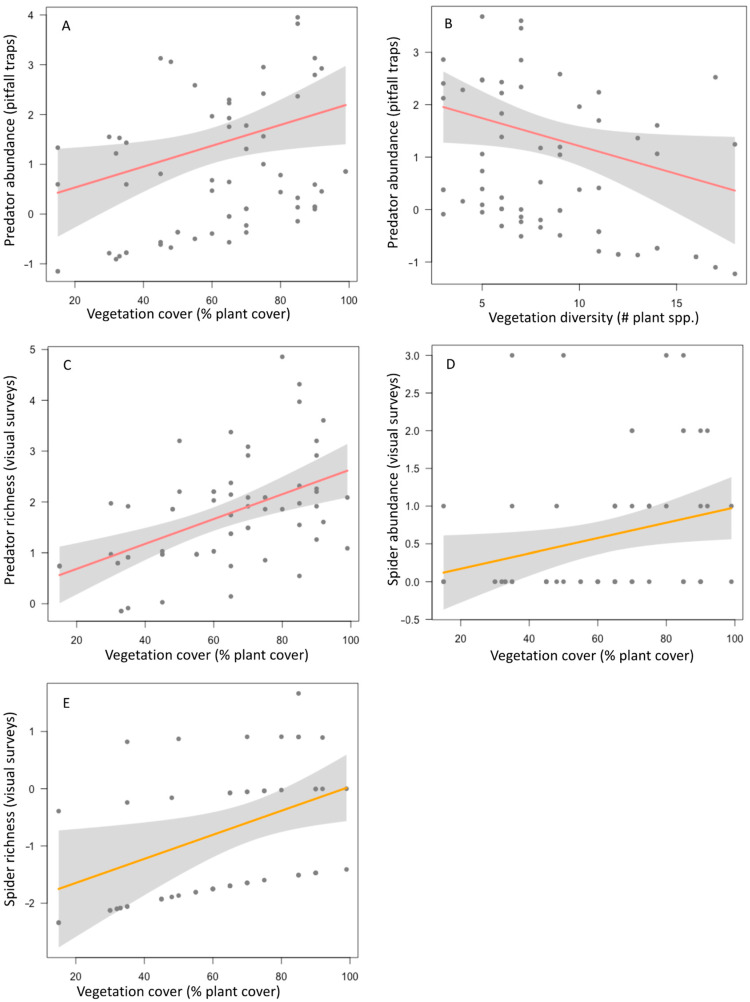
Relationships between significant vegetation complexity factors and the abundance and species richness of all predators (**A**−**C**) and spiders (**D**,**E**). Each dot represents a sampled plot at community garden sites in the California central coast. Lines show the fitted models, and gray shading indicates model confidence bands (95% confidence interval).

Herbivorous arthropods did not show a significant response to any of the vegetation complexity factors considered here, while predators consistently had positive associations with vegetation cover in garden plots (Table 1). Plots with high amounts of vegetation cover supported more foliage-dwelling predator morphospecies as well as a higher abundance of ground-dwelling predators (Figure 1A,B). Yet, plots with high vegetation diversity had fewer ground-dwelling predators (Figure 1C). Plots with high vegetation cover had a higher abundance of spiders and more spider morphospecies, but ants did not respond to any vegetation complexity metric (Figure 1D,E).

**Figure 2:**
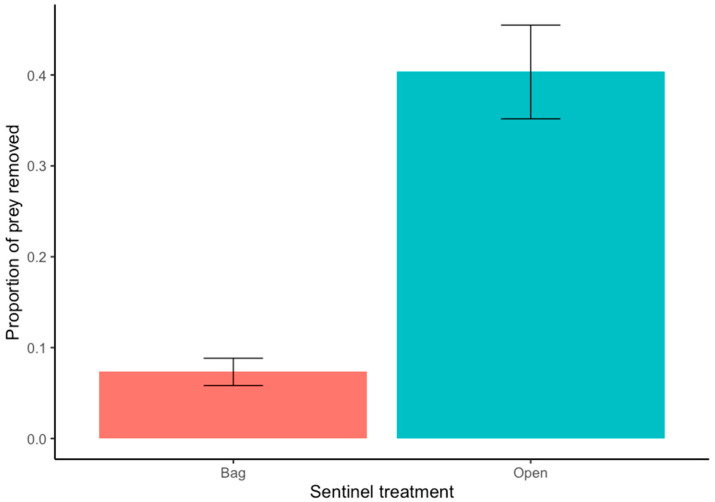
Results of sentinel pest predation experiments conducted in gardener plots at urban community gardens in the California central coast. Bars show proportions of cornworm egg prey removed from open and bagged (predator exclusion) fava bean plants after 24 h. Error bars represent one standard error. The proportion of prey removed was significantly different between open and bagged treatments for eggs (W = 2998.5, *p* = <0.001).

**Figure 3:**
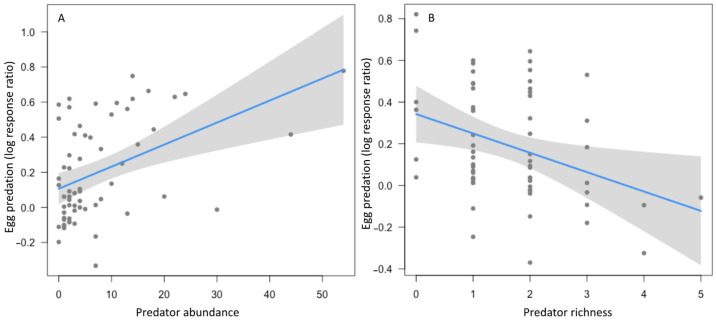
Relationships between significant predictors and egg predation in sentinel pest experiments at urban community gardens in the California central coast. Egg predation was positively associated with predator abundance (**A**) and negatively associated with the species richness of predators (**B**). Each dot represents a sampled plot at community garden sites in the California central coast. Lines show the fitted models, and gray shading indicates model confidence bands (95% confidence interval).

About 40% of egg prey were removed in open egg predation treatments. The proportion of egg prey removed was about four times higher in open compared to bagged treatments (W = 2998.5, *p* = <0.001) (Figure 2). None of the vegetation complexity factors were significant predictors of egg predation, but we found that egg predation was positively associated with predator abundance and negatively associated with predator richness (Figure 3). One qualification to our result showing a positive association between predator abundance and egg predation is that three gardener plots had much higher predator abundance due to high ant abundance in these plots (Figure 3A). 

## 4. Discussion

Overall, we found that of the three components of vegetation complexity examined (cover, diversity, and connectivity), predator abundance and richness responded to vegetation cover and diversity, while herbivore abundance and richness and egg predation did not respond to any of the measured factors. Predators removed up to 40% of egg prey (more than four times the control treatment) in sentinel egg predation experiments, revealing their important predation services in gardens. However, while higher predator abundance led to increased egg predation, higher predator richness resulted in a decline in egg predation, suggesting that antagonistic interactions between predator species may negatively impact egg predation in urban agroecosystems.

First, the vegetation complexity factors we focused on had significant positive effects on predators active on foliage. Gardener plots with high vegetation cover supported more foliage-dwelling predator morphospecies than plots with low cover. The positive effect of vegetation cover on arthropod diversity is likely related to favorable habitat conditions contributed by vegetation cover. Past work showed that more vegetation cover provides shading, cooler air temperatures, and higher moisture retention [68,69]. Vegetation cover in urban agriculture differs from vegetation cover in rural agriculture, where monocultures of a single crop type make up most of the vegetation present in fields [23,70]. In contrast, vegetation cover in urban gardens typically includes a much larger diversity of crop plants [71,72], so garden vegetation cover may contribute even greater habitat complexity compared to rural agroecosystems. The suggested importance of vegetation cover in our system aligns with a study of urban greenspaces in Australia [73], which showed strong positive responses of herbivores and predators to plant volume, while responses to plant diversity were variable and species-specific. From rural agriculture research, one study examined variation in the size of *Brassica* plant species, which affects vegetation cover, and similarly found positive associations between plant size and predator species richness [74]. Overall, our results largely agree with the findings of other studies showing positive effects of urban vegetation structure and complexity on predator abundance and species richness [42,75]. 

Further, plots with high vegetation cover supported greater spider abundance and morphospecies richness on plot foliage, while no vegetation complexity metrics significantly affected ants. Spiders were the most diverse group of predators in this study, and they include species that varied in foraging strategies, such as web builders, ambush predators, and ground hunters [76]. Most foliage-dwelling spiders sampled here were web-building spiders, for which greater vegetation cover can represent a greater availability of web attachment sites and shelter from inclement weather conditions, as well as more prey [77]. Several previous studies support positive associations between spider abundance and richness and vegetation cover and structure in urban and rural settings [44,77,78]. With respect to ants, our study aligns with the findings of several other urban ant studies that documented negative or no effects of vegetation structure on ant species richness [79,80,81,82]. Vegetation structure effects on ants can depend on factors such as ant size and morphological traits. Since ants are highly active foragers, vegetation structure has implications for energy expenditure, with complex structured habitats being less energy-efficient to navigate, especially for small ants [81]. In contrast, large ant species can be more common in complex habitats with multiple layers of vegetation due to their larger foraging areas [11]. While vegetation complexity supports a diversity of abundant garden spider species, it may not directly benefit the ant species found in our garden sites.

Regarding ground-dwelling predators, vegetation cover was positively associated with their abundance and diversity, whereas vegetation diversity was negatively associated with their abundance. The positive effects of vegetation cover on ground-foraging predators suggests that ground arthropods may benefit indirectly from vegetation cover. For instance, plots with more vegetation likely accumulate more leaf litter or correlate to other changes in ground cover and microclimate, which can influence ground arthropod community composition [83]. A larger volume and depth of leaf litter provides important microhabitats and greater prey availability that can support ground-foraging predator species, such as ground-hunting spiders [10]. Spiders, including several active, ground-hunting species, made up about half the ground predator species we sampled and may thus have a large influence on the positive effects of vegetation cover we observed. On the other hand, ants were the most abundant ground-level predator and had several nonsignificant negative associations with vegetation diversity, so it is possible that ants may be driving the negative association between the abundance of ground predators and vegetation diversity. Ants are generalist predators that can form mutualistic associations with honeydew-producing hemipterans that accumulate on plants [84]. All four ant species found in pitfall traps also occurred on plant foliage. Therefore, it is possible that ants spend more time foraging on foliage when more plant species are present in gardener plots due to greater availability of prey and mutualist species, resulting in a decline in ant abundance at the ground level. One previous study showed that Argentine ants were ten times more common on foliage when aphid mutualists were present compared to when aphids were absent [85]. Greater plant diversity and associated herbivores may thus have shifted the foraging location of ants in our study.

In contrast to our predator results, we did not find a significant effect of vegetation complexity metrics on either herbivore abundance or morphospecies richness. This result is consistent with a previous study showing no effect of garden characteristics on herbivore populations in residential gardens, community gardens, and urban farms, possibly due to low herbivore abundances in sampled sites [53]. In our study, we similarly found low herbivore numbers, with two herbivore morphospecies and fifteen herbivore individuals on average per plot. It is also possible that herbivores are responding to garden characteristics that we did not measure in this study. The herbivores sampled in this study use a range of garden crops as host plants, and the abundance of their respective host plants may be an important factor in determining herbivore abundance and richness, especially for herbivores who specialize on particular host plants [86]. Additionally, the effect of vegetation complexity is likely impacted by species traits such as herbivore diet breadth, mobility, and feeding mode [34]. Aphids and whiteflies, the two most common herbivore species in this study, differ in their mobility levels, where even winged aphids generally have low mobility and thus face challenges in navigating fragmented, heterogenous landscapes [87]. In contrast, whitefly adults are stronger fliers capable of dispersing over 5 km [88]. Major differences in mobility can alter herbivore responses to vegetation heterogeneity, with high-mobility herbivore populations being less affected by the composition and fragmentation of vegetation patches compared to lower-mobility herbivores [89].

While our study did not detect a significant effect of any of the vegetation complexity metrics on egg predation, predator abundance was positively associated with egg predation. Predators were active and removed up to 40% of prey from egg predation experiment plants within 24 h. Predators increased in abundance in response to vegetation cover in gardener plots, suggesting that vegetation complexity indirectly supports predation by supporting greater numbers of predators. One caveat to this result is that the three plots with much higher predator abundance (Figure 3A) were due to high ant abundance in these plots. As social insects, ants can be superabundant predators compared to other predators that display more solitary foraging behavior. Surges in ant abundance in these plots may be strongly influencing our result showing a positive relationship between predator abundance and egg predation. Even though high predator abundance in these three plots may appear to be outliers in the dataset, we argue that this is actually important data to include in the analysis from a biological perspective because high ant abundance can lead to high egg predation, as is shown here and in other studies [62]. Further, predator species richness was negatively associated with egg predation. Based on the species composition of predators present in gardens, one proposed explanation for this result is that intraguild predation among predator species limits egg predation. For instance, spiders are generalist predators that often engage in intraguild predation of smaller spiders and other predators [90,91], which can reduce biological control. One study of biological control in grass–clover fields manipulated the number of wolf spiders in fields and observed no impact on prey taxa when the number of wolf spiders was increased [92]. However, fewer wolf spiders elevated the abundance of other ground spiders, suggesting that competition among spider species can interfere with biological pest control. Evidence from spider molecular gut content analysis shows that intraguild predation among spiders is more common when diverse prey are unavailable and resource niches are limited [93]. Thus, low numbers of herbivores along with a diverse spider community could account for the negative impact of predator richness on egg predation observed in our study. 

Additionally, the presence of the Argentine ant (*Linepithema humile*), a species that is often aggressively invasive within its territory [94], may also play a role in limiting biological pest control by other ant species. Previous work documented that Argentine ants can reduce the foraging success of native ant species by outcompeting foraging native ants and successfully fighting off native ants from food baits [95,96]. While we did not directly observe aggression between Argentine ants and other species, high Argentine ant abundance in our study (present in ~58% of plots) suggests that antagonistic interactions likely occur and could potentially reduce biological pest control. Our study represents a brief snapshot of the arthropod community in gardens, and longer-term study would be necessary to confirm whether antagonism between predator species presents a cause for concern in the provision of biological control. Future research could clarify whether Argentine ants specifically hinder biological control in gardens.

Finally, our study took place over six days in eight community garden sites, which we recognize as a limitation of the generalizability of our results. The difficulty in replicating this intensive sampling across the region (due to site availability and time constraints) limited the number of sites and sample size of our study, so our results may not be comparable across all community gardens. Further, a snapshot study such as ours cannot elucidate longer-term patterns and processes shaping arthropod communities and predation in community gardens. However, our short-term, intensive sampling was able to pair data on garden vegetation, arthropods, and egg predation. Community gardens are dynamic systems that change rapidly as gardeners sow, harvest, and make other management changes to the plants and soil within their plots, often on a daily basis. Short-term studies can help us begin to understand the multitrophic relationships involved in applied settings [97,98]. Future studies can extend and improve this understanding.

## 5. Conclusions

This study shows that vegetation cover is an important component of vegetation complexity in community gardens, with implications for the predator community in garden plots. Garden plots with higher vegetation cover supported higher species richness of foliage-dwelling predators and higher abundance of ground-dwelling predators. Additionally, spiders were more abundant and diverse in plots with high vegetation cover. We also found a negative association between vegetation diversity and the abundance of ground-dwelling arthropods, but this result is likely driven by a shift in ant foraging activity from the ground level to foliage when vegetation diversity is high. Our results generally support the idea that structurally complex vegetation supports predator diversity and abundance, as predicted by the enemies hypothesis. In community gardens where vegetation diversity is inherently high due to the diversity of gardeners and growing practices, vegetation cover may be relatively more important to manage as a component of habitat complexity.

Despite the increases in predator abundance and diversity documented in our study, the herbivore community and egg predation were unaffected by vegetation complexity, possibly due to antagonistic interactions between predator species. Based on sentinel pest experiments, we found that gardener plots with higher predator richness had reduced predation on sentinel corn earworm eggs. Our study therefore suggests that antagonism between predator species may potentially limit predation by predators engaged in intraguild predation or interference competition. If severe antagonism persists, future research could determine ecological conditions that result in predator antagonism and possible mitigating factors. However, conserving predators in garden plots arguably benefits the entire community garden, and our study supports the idea that abundant predators can enhance pest suppression in garden plots. Overall, our results demonstrate that the decisions gardeners make about the vegetation in their garden plots have important consequences for the predators inhabiting their community gardens. Based on these results, one management recommendation gardeners can implement is to add more vegetation cover in their plots to increase structural complexity and promote recruitment of predators.

## Figures and Tables

**Table 1:** Model output from generalized linear models testing relationships between vegetation complexity metrics, herbivore and predator variables, and egg predation in urban community gardens in the California central coast. Predator sampling method is indicated by VS (visual survey) or PT (pitfall trap).

Response Variable	Model Type	No. Models	R^2^ *	Predictor Variables	No. Models with Variable	Estimate	z- or t- ** Value	*p*-Value
Herbivore abundance	Average	4	<0.001–0.05	Cover	1	−0.018	1.66	0.096
				Diversity	1	−0.091	1.36	0.173
				Connectivity	1	−11.3	0.929	0.353
Herbivore richness	Average	2	<0.001–0.01	Cover	1	0.005	0.932	0.351
Predator abundance (VS)	Best	1	<0.001	(Intercept)	NA	2.00	11.8	<0.001
Predator abundance (PT)	Average	3	<0.001–0.072	Cover	2	0.02	2.032	0.042
				Diversity	1	−0.106	1.983	0.047
Predator richness (VS)	Average	2	0.172–0.204	Cover	2	0.022	3.49	<0.001
				Diversity	1	−0.058	1.69	0.092
Predator richness (PT)	Average	5	<0.001–0.048	Cover	2	0.009	1.14	0.255
				Diversity	2	−0.063	1.37	0.172
				Connectivity	1	4.16	0.734	0.463
Ant abundance (VS)	Average	2	<0.001–0.009	Diversity	1	0.039	0.727	0.467
Ant abundance (PT)	Average	5	<0.001–0.076	Cover	2	0.021	1.87	0.062
				Diversity	2	−0.111	1.67	0.094
				Connectivity	1	8.01	0.909	0.363
Ant richness (VS)	Average	2	0.039–0.072	Cover	2	0.009	1.28	0.201
				Diversity	1	−0.045	1.07	0.285
Ant richness (PT)	Average	3	<0.001–0.016	Diversity	1	−0.042	1.07	0.286
				Connectivity	1	4.67	0.831	0.406
Spider abundance (VS)	Average	2	0.072–0.075	Cover	2	0.01	2.02	0.044
				Diversity	1	−0.017	0.511	0.61
Spider abundance (PT)	Average	5	<0.001–0.158	Cover	3	0.033	1.78	0.075
				Diversity	1	−0.136	1.47	0.143
				Connectivity	2	15.6	1.19	0.232
Spider richness (VS)	Average	5	0.092–0.01	Cover	2	0.022	2.34	0.02
				Connectivity	1	−5.06	0.616	0.538
Spider richness (PT)	Average	3	<0.001–0.111	Cover	2	0.023	1.65	0.099
				Diversity	1	−0.149	1.83	0.066
Egg predation	Average	4	0.12–0.214	Cover	2	0.003	1.52	0.128
				Diversity	1	−0.009	0.855	0.393
				Predator abundance	4	0.012	3.25	0.001
				Predator richness	3	−0.084	2.1	0.036

* Range of McFadden’s pseudo-R^2^ values is reported for averaged models. ** z-value for averaged models, t-value for best models.

## Data Availability

The summary data used in this study are available on Dryad at https://doi.org/10.5061/dryad.wpzgmsbvj (accessed on 14 November 2023).

## Supplementary Materials

The following supporting information can be downloaded at: https://www.mdpi.com/article/10.3390/insects15010041/s1, Table S1: Pearson correlation matrix of vegetation complexity variables; Table S2: Predator and herbivore morphospecies; Table S3: Model output from generalized linear mixed models (including site as a random effect) testing relation-ships between vegetation complexity metrics, herbivore and predator variables, and egg predation in urban com-munity gardens in the California central coast.
