# Supplementary material for: Local Habitat Complexity and Its Effects on Herbivores and Predators in Urban Agroecosystems
Source: Insects. 2024 Jan 7;15(1):41. doi: 10.3390/insects15010041 (PMC10816164; doi:10.3390/insects15010041)
Supplement: Supplementary file 1 [file insects-15-00041-s001.zip › insects-2748864-supplementary.pdf]

insects

Article

Local Habitat Complexity and Its Effects on Herbivores and Predators in Urban Agroecosystems

Azucena Lucatero[1,*], Shalene Jha[2,3], and Stacy M. Philpott[1]

[1] Environmental Studies Department, University of California, Santa Cruz, CA 95064, USA
[2] Integrative Biology Department, University of Texas at Austin, Austin, TX 78712, USA
[3] Lady Bird Johnson Wildflower Center, University of Texas at Austin, Austin, TX 78739, USA
* Correspondence: alucater@ucsc.edu

Supplementary Material

Table S1. Pearson correlation matrix of vegetation complexity variables, gardener plot size, and predator abundance and richness in urban community garden plots (*n* = 64) in the California central coast. Values in cells show correlation coefficients, and asterisks show significance.

	Density	Diversity	Connectivity	Gardener plot size	Predator abundance	Predator richness
Cover	-	0.55*	0.44*	0.04	-0.01	0.43*
Diversity	0.55*	-	0.10	-0.01	0.04	0.07
Connectivity	0.44*	0.1	-	-0.12	0.02	0.17
Gardener plot size	0.04	-0.01	-0.12	-	0	-0.12
Predator abundance	-0.01	0.04	0.02	0	-	0.3*
Predator richness	0.43*	0.07	0.17	-0.12	0.3*	-

* $p < 0.05$

Table S2. Natural enemy and herbivore morphospecies observed in visual surveys and pitfall traps deployed in urban community gardens in the California central coast.

Trophic group	Order	Family	Morphospecies	Visual survey count	Pitfall trap count
Predators	Araneae	Anyphaenidae	*Anyphaena sp. 1*	3	3
		Clubionidae	*Clubionidae sp. 1*	5	0
		Dysderidae	*Dysdera crocata*	0	4
		Gnaphosidae	*Scotophaeus blackwalli*	0	4
		Linyphiidae	*Erigone sp. 1*	3	0
			Lyniphiidae sp. 1	0	1
			Microlinyphia sp. 1	3	1
			Neriene dana	0	2
			Neriene digna	0	1
		Lycosidae	*Arctosa sp. 1*	0	10
			Pardosa sierra	2	0
			Pardosa sp. 1	1	7
		Salticidae	*Saliticae sp. 1*	2	0
		Tetragnathidae	*Tetragnatha sp. 1*	3	0
		Theridiidae	*Enoplognatha ovata*	1	0
			Latrodectus hesperus	2	0
			Neriene sp. 1	2	0
			Parasteatoda/Cryptachaea sp. 1	4	0
			Steatoda nobilis	2	0
			Steatoda sp. 1	2	0
			Theridion sp.1	1	0
		Zoropsidae	*Titiotus sp. 1*	0	2
			Titiotus sp. 2	0	1
	Opiliones		*Opiliones UnkGenSp*	6	0
	Coleoptera	Carabidae	*Carabidae sp. 1*	0	2
			Laemustenus contemplanus	0	1
			Scaphinotus sp. 1	0	1
		Staphylinidae	*Staphylinidae sp. 1*	2	25
			Sunius sp. 1	1	0
		Tenebrionidae	*Coelocnemis californica*	0	1
	Diptera	Dolichopodidae	*Dolichopidae sp. 1*	12	0
		Mecoptera	*Mecoptera sp. 1*	1	0
		Syrphidae	*Syrphidae sp. 1*	47	0
	Hemiptera	Anthocoridae	*Anthocoridae sp. 1*	1	0
		Geocoridae	*Geocoris sp. 1*	1	6
		Nabidae	*Nabis sp. 1*	1	1

	Hymenoptera	Formicidae	*Cardiocondyla mauritanica*	2	10
			Hypoponera opacior	1	6
			Linepithema humile	311	112
			Monomorium ergatogyna	34	53
			Prenolepis imparis	3	0
			Tetramorium sp. 1	1	0
		Mymaridae	*Mymaridae sp. 1*	1	0
		Platygastridae	*Platygastridae sp. 1*	1	0
			TOTAL	462	254
Herbivores	Coleoptera	Chrysomelidae	*Acalymma vittatum*	3	NA
			Chrysomelidae UnkGenSp	4	NA
			Diabrotica sp. 1	2	NA
			Epitrix hirtipennis	2	NA
		Ptinidae	*Stegobium paniceum*	1	NA
		Scraptidae	*Scraptidae UnkGenSp*	1	NA
	Diptera	Sciarcidae	*Sciarcidae UnkGenSp*	2	NA
	Hemiptera	Aleyrodidae	*Bemisia tabaci*	113	NA
		Aphididae	*Acyrthosiphon pisum*	94	NA
			Aphis fabae	154	NA
			Brevicoryne brassicae	72	NA
			Myzus persicae	1	NA
		Cicadellidae	*Empoasca sp. 1*	12	NA
			Euscelidius sp. 1	4	NA
		Miridae	*Miridae UnkGenSp*	20	NA
	Lepidoptera	Pieridae	*Pieris rapae*	14	NA
	Trombidiformes	Tetranychidae	*Tetrachynus sp. 1*	3	NA
			TOTAL	502	NA

Table S3. Model output from generalized linear mixed models (including site as a random effect) testing relationships between vegetation complexity metrics, herbivore and predator variables, and egg predation in urban community gardens in the California central coast. Predator sampling method is indicated by VS (visual survey) or PT (pitfall trap). We did not include these models in the main text because of model convergence and overfitting issues.

Response variable	Model type	No. models	Predictor variables	No. models with variable	Estimate	z or t* value	p-value
Herbivore abundance	Average	6	Cover	2	-0.004	-0.004	0.575
			Diversity	3	-0.065	2.11	0.035
			Connectivity	1	-0.85	0.074	0.9409
Herbivore richness	Average	4	Cover	3	0.006	1.01	0.314
			Diversity	2	0.001	0.002	0.999

			Connectivity	1	-1.87	0.374	0.709
Predator abundance (VS)	Average	4	Cover	2	0.001	0.356	0.722
			Diversity	3	0.005	0.371	0.711
			Connectivity	1	1.31	0.345	0.73
Predator abundance (PT)	Average	2	Cover	2	0.021	2.54	0.011
			Diversity	3	-0.106	2.45	0.014
			Connectivity	1	1.15	0.154	0.878
Predator richness (VS)	Average	4	Cover	2	0.002	0.754	0.451
			Diversity	3	-0.027	1.71	0.088
			Connectivity	1	0.807	0.274	0.784
Predator richness (PT)	Average	5	Cover	2	0.009	1.02	0.309
			Diversity	3	-0.062	1.23	0.217
			Connectivity	1	4.16	0.658	0.51
Ant abundance (VS)	Average	4	Cover	2	0.001	0.346	0.73
			Diversity	3	0.005	0.096	0.924
			Connectivity	1	-1.54	199	<0.001
Ant abundance (PT)	Average	2	Cover	2	0.02	2.24	0.025
			Diversity	3	-0.12	2.46	0.014
			Connectivity	1	9.6	1	0.316
Ant richness (VS)	Average	5	Cover	2	0.009	1.28	0.202
			Diversity	3	-0.029	0.685	0.493
			Connectivity	1	5.21	0.968	0.333
Ant richness (PT)	Average	5	Cover	2	-0.001	0.236	0.813
			Diversity	3	-0.043	1.09	0.276
			Connectivity	1	4.85	0.867	0.386
Spider abundance (VS)	Average	3	Cover	2	0.02	1.84	0.066
			Diversity	3	-0.017	0.277	0.781
			Connectivity	1	-1.59	0.153	0.879
Spider abundance (PT)	Average	3	Cover	2	0.037	2.31	0.021
			Diversity	3	-0.162	1.71	0.087
			Connectivity	1	-6.01	0.361	0.718
Spider richness (VS)	Average	3	Cover	2	0.024	8.17	<0.001
			Diversity	3	0.003	0.73	0.465
			Connectivity	1	-4.81	1045	<0.001
Spider richness (PT)	Average	4	Cover	2	0.025	1.61	0.107
			Diversity	3	-0.146	1.6	0.109
			Connectivity	1	-8.2	0.496	0.62
Egg predation	Average	3	Connectivity	2	-0.99	0.618	0.536
			Predator	1	0.009	3.18	0.001

	abundance
